# Transcriptomic and Proteomic Analysis of Clear Cell Foci (CCF) in the Human Non-Cirrhotic Liver Identifies Several Differentially Expressed Genes and Proteins with Functions in Cancer Cell Biology and Glycogen Metabolism

**DOI:** 10.3390/molecules25184141

**Published:** 2020-09-10

**Authors:** Christoph Metzendorf, Katharina Wineberger, Jenny Rausch, Antonio Cigliano, Kristin Peters, Baodong Sun, Daniela Mennerich, Thomas Kietzmann, Diego F. Calvisi, Frank Dombrowski, Silvia Ribback

**Affiliations:** 1 Institut fuer Pathologie, Universitaetsmedizin Greifswald, Friedrich-Loeffler-Str. 23e, 17475 Greifswald, Germany; christoph.metzendorf@igp.uu.se (C.M.); katharina.wineberger@hotmail.de (K.W.); jesora@web.de (J.R.); antonio.cigliano@klinik.uni-regensburg.de (A.C.); kristin.peters@med.uni-greifswald.de (K.P.); diego.calvisi@klinik.uni-regensburg.de (D.F.C.); frank.dombrowski@uni-greifswald.de (F.D.); 2 Division of Medical Genetics, Department of Pediatrics, Duke University Medical Center, Durham, NC 27710, USA; baodong.sun@duke.edu; 3 Faculty of Biochemistry and Molecular Medicine, University of Oulu, 90570 Oulu, Finland; daniela.mennerich@oulu.fi (D.M.); thomas.kietzmann@oulu.fi (T.K.); 4 Biocenter Oulu, University of Oulu, 90570 Oulu, Finland

**Keywords:** clear cell foci, liver, hepatocellular carcinoma, pre-neoplastic lesions

## Abstract

Clear cell foci (CCF) of the liver are considered to be pre-neoplastic lesions of hepatocellular adenomas and carcinomas. They are hallmarked by glycogen overload and activation of AKT (v-akt murine thymoma viral oncogene homolog)/mTOR (mammalian target of rapamycin)-signaling. Here, we report the transcriptome and proteome of CCF extracted from human liver biopsies by laser capture microdissection. We found 14 genes and 22 proteins differentially expressed in CCF and the majority of these were expressed at lower levels in CCF. Using immunohistochemistry, the reduced expressions of STBD1 (starch-binding domain-containing protein 1), USP28 (ubiquitin-specific peptidase 28), monad/WDR92 (WD repeat domain 92), CYB5B (Cytochrome b5 type B), and HSPE1 (10 kDa heat shock protein, mitochondrial) were validated in CCF in independent specimens. Knockout of *Stbd1*, the gene coding for Starch-binding domain-containing protein 1, in mice did not have a significant effect on liver glycogen levels, indicating that additional factors are required for glycogen overload in CCF. *Usp28* knockout mice did not show changes in glycogen storage in diethylnitrosamine-induced liver carcinoma, demonstrating that CCF are distinct from this type of cancer model, despite the decreased USP28 expression. Moreover, our data indicates that decreased USP28 expression is a novel factor contributing to the pre-neoplastic character of CCF. In summary, our work identifies several novel and unexpected candidates that are differentially expressed in CCF and that have functions in glycogen metabolism and tumorigenesis.

## 1. Introduction

The early processes underlying human hepatocellular carcinogenesis are poorly understood. Very diverse conditions, such as the cirrhotic liver, non-cirrhotic liver with glycogen storage disease type I [1], as well as metabolic disorders (alpha-1-antitrypsin deficiency and hemochromatosis), obesity [2], hyperinsulinism, alcohol abuse, and type 2 diabetes mellitus [3,4], are known to be risk factors for hepatocellular carcinoma (HCC) development. While high-grade dysplastic nodules in liver cirrhosis are accepted as pre-neoplastic lesions of HCC [5], the situation in the absence of liver cirrhosis is less clear, even though 15–20% of HCC occurs in non-cirrhotic livers [6].

To better understand the mechanisms underlying carcinogenesis, it is important to better characterize the precursor stages—pre-neoplastic lesions—for improving early diagnosis and treatment of HCC. This is increasingly important as primary liver cancer is the fifth most frequent malignancy worldwide and the proportion of HCCs in the background of type 2 diabetes and obesity is becoming more common [2].

In the human cirrhotic liver, different types of foci of altered hepatocytes were described by Bannasch: glycogen-storing foci (clear cell foci (CCF), with pale hematoxylin and eosin (HE) staining), mixed cell foci, and basophilic foci [7]. These foci are also well known in diverse animal models of hepatocarcinogenesis [8] and their progression to hepatocellular adenomas and HCC is well described [7,9,10,11]. Moreover, using the intraportal pancreatic islet transplantation model of hepatocarcinogenesis [12,13,14,15], we found the AKT (v-akt murine thymoma viral oncogene homolog)/mTOR (mammalian target of rapamycin) and the Ras (rat sarcoma)/MAPK (mitogen-activated protein kinase) pathways to be activated throughout the development of CCF to HCC, where they play important roles as major oncogenic downstream effectors of insulin signaling [16,17]. The lipogenic phenotype is characterized by increased lipogenesis and storage of lipid droplets. These alterations have also been described in human HCC, where they are associated with unfavorable prognosis [18,19]. Recently, we described that CCF in human non-cirrhotic livers reveal many molecular and metabolic characteristics, like pre-neoplastic liver foci of the hormonal model of hepatocarcinogenesis after intraportal pancreatic islet transplantation [20]. Specifically, we found an increase in glycogen storage, reduced glucose-6-phosphatase activity, and an upregulation of enzymes regulating glycolysis, de novo lipogenesis, and beta-oxidation, as well as overexpression of the insulin receptor and activated AKT/mTOR and Ras/MAPK pathways in CCF from both human livers and the rat model [20]. Similarly, in the mouse, hepatocarcinogenesis is associated with activation of the insulin/AKT/mTOR signaling pathway, the transcriptional regulator ChREBP (Carbohydrate-response element-binding protein) [16,21], as well as the lipogenic pathway [18,22,23].

Although these data hint to several pathways and regulators, a comprehensive inventory of gene and protein expression in human CCF is missing.

In the current work, we applied microarray analysis and proteomics after laser microdissection as unbiased approaches to identify and further characterize CCF of human non-cirrhotic liver parenchyma. To this end, we compared RNA and protein expression in CCF with neighboring tissue as a control and found several genes and proteins with significantly altered expression. Starch-binding domain-containing protein 1 (STBD1), ubiquitin-specific peptidase 28 (USP28), WD repeat-containing protein 92 (WDR92)/Monad, and heat shock protein family E (Hsp10) member 1 (HSP10) were among the candidates with the highest differential expression, and their expression was validated by immunohistochemistry.

## 2. Results

### 2.1. More RNAs Have Reduced Expression in CCF Compared to Controls

After standard processing of the microarray dataset, as described in the Materials and Methods Section, we performed the following tests to identify any problems with sample quality, normalization, or signal quality. First, we determined how many transcripts were above the non-detection threshold in each sample and did not identify any samples to exclude (Supplementary Figure S1A). Also, the mean number of transcripts of control and CCF samples did not show any statistically significant differences (Supplementary Figure S1A,B; *n* = 18, Student’s *t*-test, *p* (unpaired) = 0.104; *p* (paired) = 0.063). The distributions of log-transformed signal intensities per sample were quite similar between samples (Supplementary Figure S2). From these observations, we concluded that the quality of the data was acceptable for further analysis.

**Figure 1:**
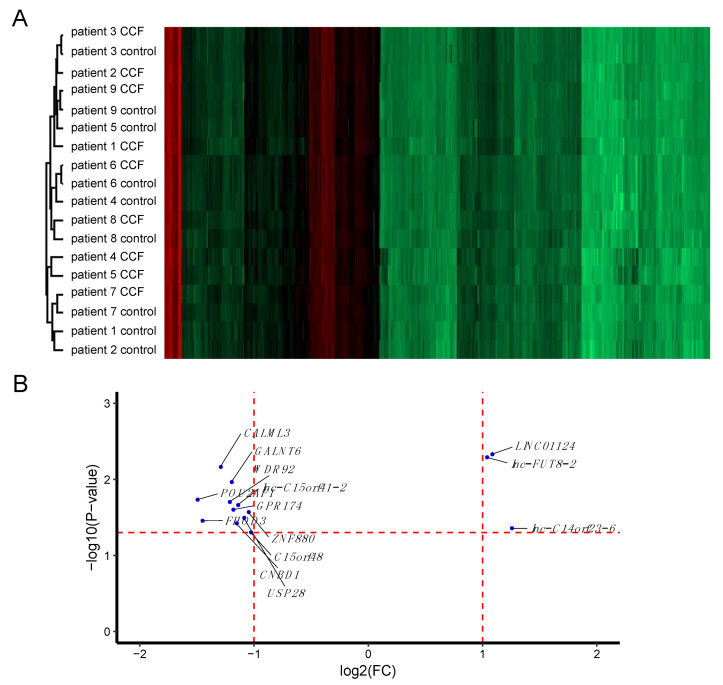
The transcriptome of CCF (clear cell foci) and control samples from human liver biopsy specimens. Control and CCF samples were laser-capture micro-dissected from cryosections and total RNA was used for microarray analysis. (**A**) Cluster analysis of the full dataset revealed larger inter-patient differences than differences between CCF and control samples. (**B**) Scatter plot of transcripts (mRNAs, miRNAs, and lncRNAs) with statistically significant differential expression in CCF vs. control samples. More transcripts show lower expression in CCF than in control samples. Horizontal red line: *p-*value = 0.05; vertical red lines: FC = 2-fold down- or up-regulation, respectively.

Using cluster analysis, samples clustered in a patient-dependent manner in most cases (Figure 1A), indicating that differences between patients were more extensive than those between CCF and control samples. A larger degree of heterogeneity is not uncommon for human tissue samples, in general, and we suggest that transcripts with statistically significant differences between CCF and control samples are likely to be quite robust. On the other hand, we may miss differentially expressed genes due to the higher degree of noise in the data. Taking the inter-patient heterogeneity into account, we compared RNA expression between CCF and controls by calculating fold-changes (CCF/control) per patient. Fourteen transcripts (Table 1 and Figure 1B) had at least 2-fold higher or lower expression in CCF than in control samples, three of these with higher and 11 with lower expression in CCF samples. Interestingly, all three transcripts with increased expression in CCF coded for long non-coding RNAs (lnc-FOXG1-6:17, LINC01124:6, and LINC02290:27). However, very little is known about the function of any of these lncRNAs. LINC01124:6 is annotated as a bidirectional, 2129 bp long lncRNA encoded by one exon, while LNC02290:27 is an intergenic lncRNA of 463 bp length encoded on 4 exons (LNCIPEDIA v 5.2, www.lncipedia.org).

Ten of the eleven genes with lower expression in CCF than in control samples code for protein-coding RNAs. The gene with the lowest expression was *POU2AF1* (encoding POU class 2 associating factor 1), which is important for the regulation of B-cell maturation [40]. The other downregulated genes have diverse functions in actin binding (FOHD3), calcium-signaling (CALML3), prefoldin-like complex and signaling components (monad/WDR92), protein *N*-acetylgalactosaminyl transfer (GALNT6), G protein-coupled receptor-mediated signaling (GPR174), nucleotide binding (CNBD1), protein de-ubiquitination (USP28), and as yet unidentified functions (C15orf48 and ZNF880 and the long non-coding RNA lnc-C15orf41-2:1) (Table 1).

### 2.2. More Proteins Have Reduced Expression in CCF Compared to Control Samples

Using mass-spectrometric analysis, we obtained quantitative data of 995 to 2253 proteins in 14 samples (seven control + seven CCF samples from the same patients), with an average of 1474 proteins identified per sample. A total of 504 proteins was identified in all 14 samples (Supplementary Figure S3A). The average number of identified proteins in control and CCF samples did not differ (means/geometric means: 1583/1559 and 1365/1315 proteins; control and CCF, respectively. Supplementary Figure S3B; Student’s *t*-test, *p* (unpaired) = 0.300). Also, there was no statistically significant difference between the number of identified proteins from CCF and control samples per patient (Supplementary Figure S3C; paired Student’s *t*-test, *p* = 0.137). The normalized log2-transformed expression data approximated normal distribution in all samples (Supplementary Figure S4).

**Figure 2:**
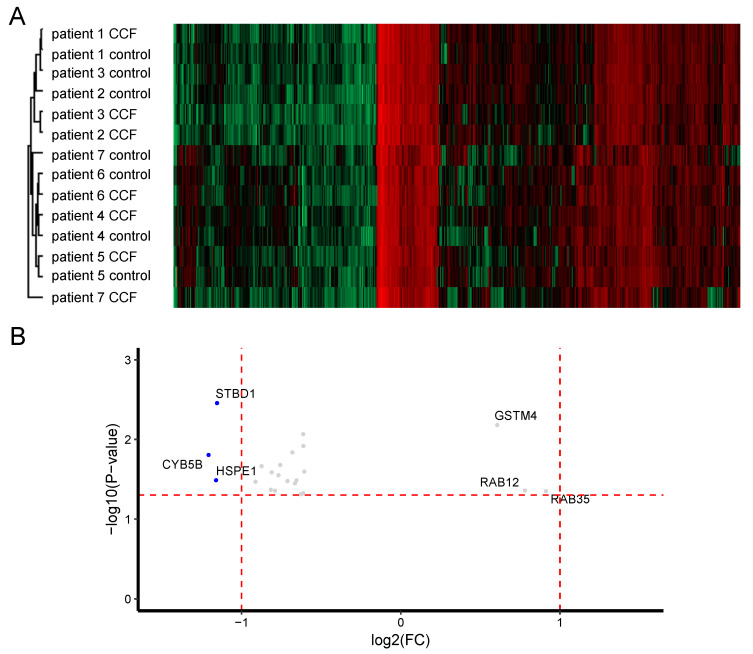
The proteome of CCF and control samples from human liver biopsy specimens. Control and CCF samples were laser-capture micro-dissected from cryosections and protein expression was quantified by LC-MS/MS. (**A**) Cluster analysis of the full dataset revealed larger inter-patient differences than differences between CCF and control samples. (**B**) Scatter plot of proteins (detection in ≥ 8 samples) with statistically significant differential expression in CCF vs. control samples. More proteins are less abundant in CCF than in control samples. Horizontal red line: *p*-value = 0.05; vertical red lines: FC = 2-fold down or upregulation, respectively. Proteins significantly different at least 1.5-fold (grey dots) and 2-fold (blue dots) between CCF and control.

As in the cluster analysis of the microarray data, cluster analysis of the proteomic data could not clearly separate control and CCF samples into distinct groups (Figure 2A). Therefore, the higher heterogeneity between patients than between CCF and control samples was also present at the protein level. Furthermore, more proteins were expressed at a lower level in CCF compared to control samples (Figure 2B and Table 2). The expression of three proteins was decreased more than 2-fold: these were Cytochrome b5 type b (CYB5B, 2.3-fold lower), mitochondrial 10 kDa heat shock protein (HSP10/HSPE1; 2.2-fold lower), and starch-binding domain-containing protein 1 (STBD1; 2.2-fold lower). Further, 19 proteins showed a downregulation of more than 1.5-fold (Figure 2B and Table 2). In contrast, there were only three proteins with more than 1.5-fold increased expression: these were, GSTM4, RAB12, and RAB35. No proteins with >= 2-fold higher expression in CCF than in controls were identified.

### 2.3. Immunohistochemical Validation of Differential Expression of Monad/WDR92, USP28, STBD1, CYB5B, and HSPE1 in CCF

**Figure 3:**
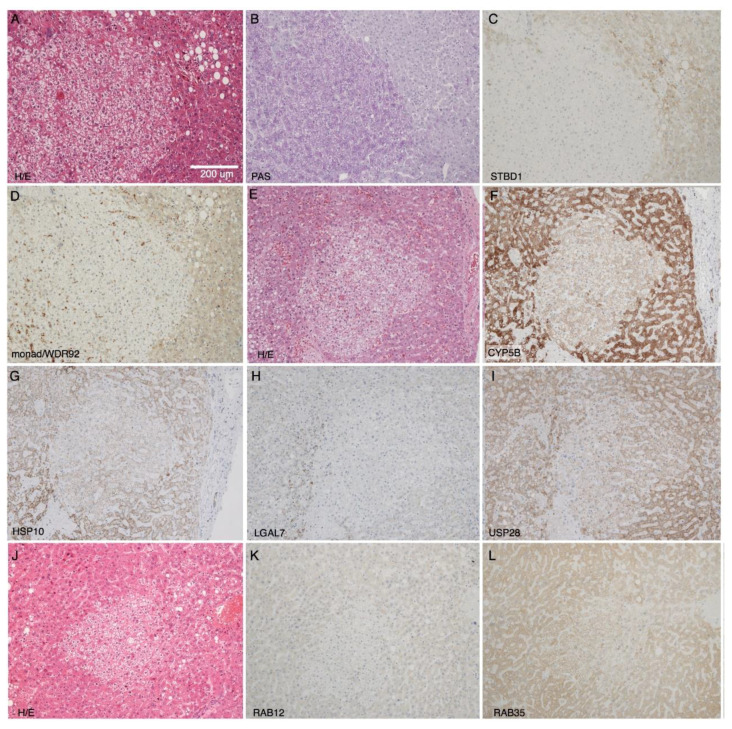
Histochemistry and immunohistochemistry of representative human liver specimens with CCF and neighboring tissue. Hematoxylin/Eosin (H/E) staining (**A**,**E**,**J**), PAS (Periodic acid-Schiff) reaction (**B**), and immunohistochemical detection of STBD1 (**C**), monad/WDR92 (**D**), CYP5B (**F**), HSP10 (**G**), LGAL7 (**H**), USP28 (**I**), RAB12 (**K**), and RAB35 (**L**). The scale bar in (**A**) is applicable to all panels (**B**–**L**).

To validate data obtained with these high-throughput methods, the protein expression of two microarray candidates (monad/WDR92 and USP28) and five proteomics candidates (the downregulated STBD1, CYB5B, and HSPE1, as well as the two upregulated RAB12 and RAB35) was analyzed in liver sections containing CCF using immunohistochemistry. Reduced expression of monad/WDR92, USP28, STBD1, CYB5B, and HSPE1 in CCF was confirmed (Figure 3 and Table 3). Increased expression of RAB12 and RAB35 in CCF could not be verified, as in most samples there was either no difference between CCF and the surrounding tissue, or a slightly lower expression in CCF (Table 3 and Figure 3). As negative controls, we used proteins with unchanged expression, like TRAP1, Cullin-3, ACSL4, COPS7A, and A-Raf. These proteins had fold changes close to one (1.37, 1.25, 1.11, 1.03, and 1.02, respectively) in the proteomic dataset. Immunohistochemical analysis confirmed no clear difference in expression of these proteins when comparing CCF to surrounding tissue (specimens from 12–15 patients analyzed, Supplementary Figure S5).

From these results, we conclude that the proteomic data from laser-capture micro-dissected samples reflects the protein composition of CCF and surrounding tissue. Our immunohistochemical data of WDR92/monad and USP28 indicate that the observed differences in protein expression in CCF and surrounding tissue could be due to regulation at the transcript level, as the mRNAs coding for these proteins were reduced in CCF according to microarray analysis.

### 2.4. Loss of STBD1 in Mice Does Not Cause Glycogen Accumulation in the Liver

Starch-binding domain-containing protein 1 (STBD1) is N-terminally anchored within the membrane of the endoplasmic reticulum and binds glycogen via its C-terminally located family 20 starch binding module [41]. An Atg8 family interacting motif (AIM) and interaction with GABARAPL1 (Gamma-aminobutyric acid receptor-associated protein-like 1) implicated STBD1 to be involved in autophagic glycogen degradation, so called glycophagy [42]. In a mouse model of Pompe disease, deletion of STBD1 suppressed lysosomal glycogen accumulation, suggesting STDB1 to be involved in transfer of glycogen from the cytoplasm to lysosomes [43].

**Figure 4:**
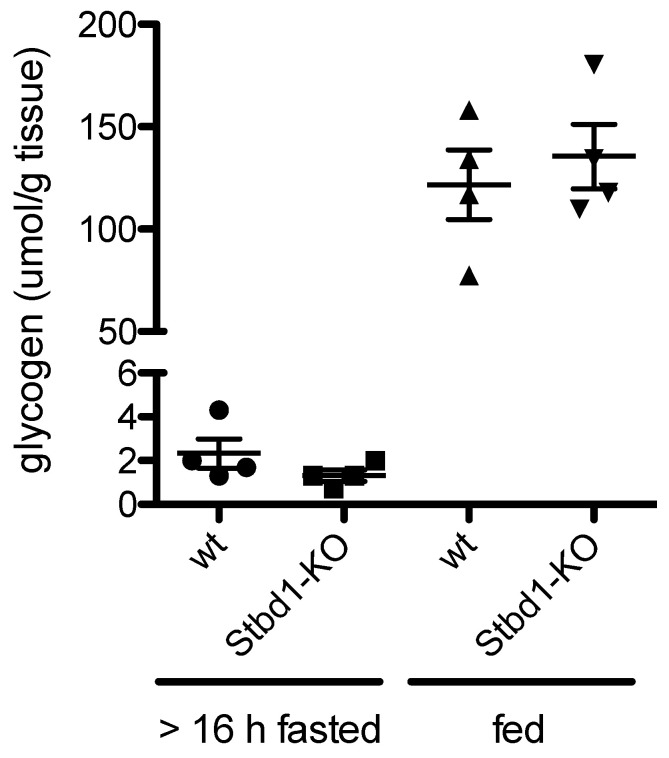
Glycogen concentrations in livers of *Stbd1*-knock-out mice after more than 16 h of fasting or in the fed state. Male mice (9 months of age) had access to food ad libitum (fed). The fasted group did not have access to food for 16 h prior to sampling. N = 4 per group, Student’s *t*-test was not significant.

The reduced expression of STBD1 in CCF observed in the present study could contribute to the hepatocellular glycogen accumulation. To test whether loss of STBD1 would result in glycogen accumulation in the liver, glycogen concentrations were determined in livers of nine-month-old male wild-type (WT) and *Stbd1*-knock-out (KO) mice with access to food throughout or fasted for 16 h prior to sacrifice. Fasting resulted in significant reductions of glycogen in bot wild type (WT) and *Stbd1*-KO mice (Figure 4). However, there was no significant difference between WT and *Stbd1*-KO mice at either condition (Figure 4). From these results, we conclude that under fasting and normal conditions, loss of STBD1 does not have a significant effect on glycogen degradation in otherwise healthy mice.

### 2.5. Diethylnitrosamine (DEN)-Induced Hepatocellular Carcinomas in Usp28-KO Mice Do Not Accumulate More Glycogen than in Control Mice

**Figure 5:**
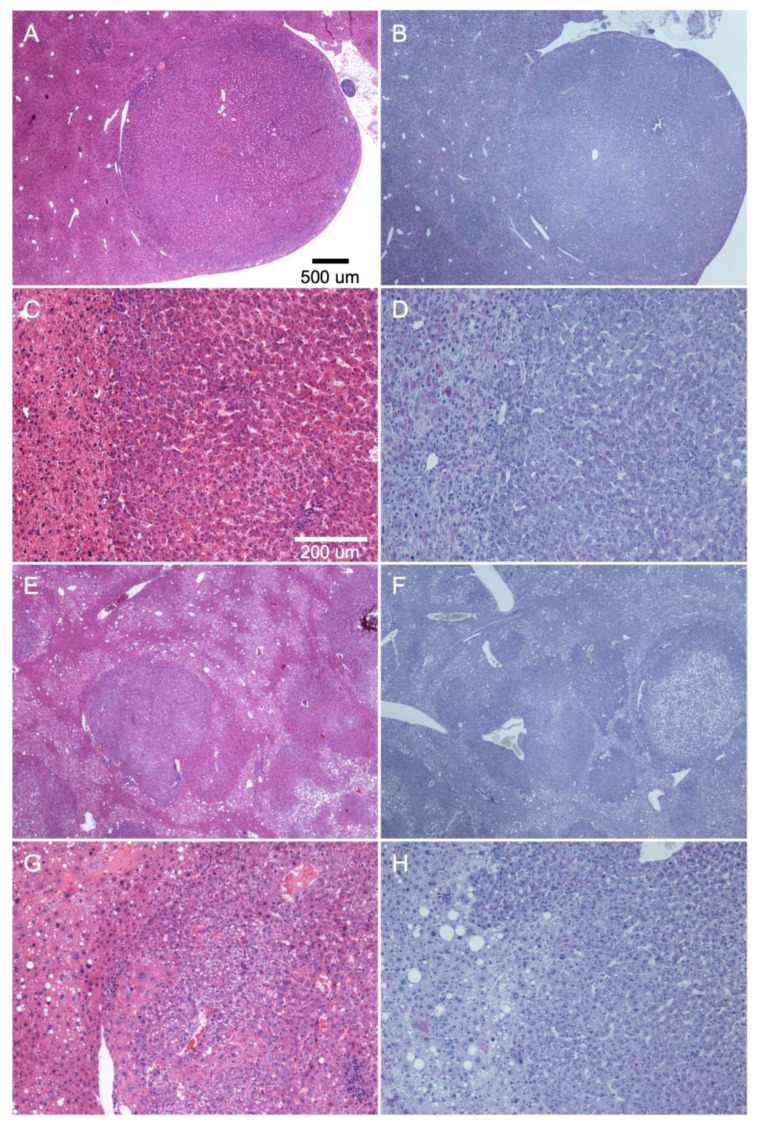
Histochemistry of diethylnitrosamine (DEN)-induced hepatocellular carcinomas of wild-type (**A**–**D**) and *Usp28* knock-out (**E**–**H**) mice. H/E stain (**A**,**C**,**E**,**G**) and PAS reaction (**B**,**D**,**F**,**H**). Representative images are shown. (**A**,**B**) and (**E**,**F**) are overviews of (**C**,**D**) and (**G**,**H**) respectively. The scale bars in (**A**) and (**C**) are applicable to (**B**,**E**,**F**) and (**D**,**G**,**H**), respectively.

Knockout of *Usp28* in mice promotes liver carcinogenesis in diethylnitrosamine (DEN)-injected mice. Although the mechanism has been reported to involve p53 through 53BP1 [44,45,46], no major impact for this regulatory axis was found in the DEN-induced HCCs of *Usp28*-KO mice [25]. As USP28 levels were downregulated in the CCF, it could mediate its effects at least partially via glycogen regulation. To investigate whether loss of USP28 may affect glycogen metabolism in carcinoma, we compared glycogen accumulation in DEN-induced HCC of WT and *Usp28*-KO mice using Periodic acid-Schiff (PAS) staining. However, no difference in PAS staining could be detected, suggesting that lack of USP28 does not cause glycogen differences in CCF (Figure 5).

## 3. Discussion

In this study, we identified novel genes and proteins that are differentially regulated in human CCF in comparison to the surrounding liver tissue to better understand the processes leading to glycogen accumulation and the possible tumor development. We found only a small number of genes/proteins with significant changes higher than two-fold. The biggest challenge was the heterogeneity of the samples, which may be due to e.g. different origins of tumors, patient medication, dietary status or age. Although desirable, it was not feasible to further increase the number of samples, as the number of appropriate specimens is limited. Furthermore, laser-capture micro-dissection of CCF is very labor-intensive: for each specimen, about 90 CCF and control samples had to be excised to obtain sufficient material for RNA and protein extraction. Hence, even though the statistical support of the omics data is not very strong and the datasets show high degrees of heterogeneity, the top scoring candidates do seem to be robust, as we were able to validate the differential expression of several candidates by immunohistochemistry using specimens from independent patient material.

Comparing the microarray and the proteomics data, no protein was found for which the respective RNA was changed accordingly. Therefore, these proteins may be not regulated at the transcript level to the same degree that they are regulated at the protein level. Since the degree of correlation between RNA level and protein expression at the genomic level is estimated to be 40–50% [47], the identified proteins may belong to the 50–60% of proteins that are mainly regulated at the post-transcriptional level.

Notably, in both the RNA and protein datasets, more genes/proteins were downregulated than upregulated in CCF, and the ratios of upregulated versus downregulated hits were similar (RNA: 3 up, 11 down (FC cutoff 2-fold), protein: 6 up, 22 down (FC cutoff 1.5-fold)). This could indicate a general non-specific replacement of cellular components by glycogen. However, we did not find indications that signal intensities of RNA and proteins were generally lower in CCF samples compared with control samples. Hence, we conclude that the differences in RNA and protein levels we observed between CCF and surrounding tissue were due to regulatory processes within cells.

### 3.1. Non-Coding RNAs

We found several non-coding RNAs to be differentially expressed in CCFs compared to controls. Unfortunately, no function is known for any of these. However, lnc-FOXG1-6:17 is an antisense lncRNA of *PRKD1* encoded by two exons (LNCIPEDIA v 5.2, www.lncipedia.org). According to current understanding, antisense transcripts are implicated in gene regulation, which can act in *cis* through transcriptional interference, double-stranded RNA/RNA masking, double-stranded RNA/RNA adenine to inosine editing, or double-stranded RNA/RNA interference. *Cis* and *trans* antisense lncRNAs may affect gene expression more globally through chromatin modifications (reviewed in Reference [48]). Hence, it is not clear if and how lnc-FOXG1-6:17 would affect the expression of *PRKD1*, which codes for Serine/threonine-protein kinase D1 (PRKD1). PRKD1 itself is implicated in cell proliferation, cell motility, invasion, protein transport, and apoptosis [49], and would represent an interesting candidate in regard to tumor development and progression in CCF. Its RNA and protein expression, though, is low in liver when compared to other tissues (human protein atlas [50,51,52]), and its mRNA level was not altered, according to our microarray analysis. Hence, a direct and strong effect of lnc-FOXG1-6:17 on *PRKD1* expression seems unlikely.

### 3.2. STBD1

Glycogen accumulation in CCF is usually explained by decreased gluconeogenesis due to lower activity of the glucose-6-phosphatase-increased glycogen synthesis and reduced glycogen degradation mediated by insulin/AKT-signaling [7]. In our current work, we observed reduced expression of starch-binding domain-containing protein 1 (STBD1) in CCF compared to control/surrounding tissue in the proteomic dataset and validated this finding by immunohistochemistry. STBD1 is a N-terminally membrane-anchored [53] glycogen-binding [41,54] protein that localizes glycogen to perinuclear sites/ER, late endosomes, and lysosomes, and is most abundantly expressed in muscle and liver [41]. The lower expression of STBD1 that we observed in CCF together with its involvement in the lysosomal glycogen degradation pathway [43] suggests that this reduction of STBD1 expression could be an additional factor contributing to glycogen accumulation in CCF. However, a significant glycogen accumulation in the livers of *Stbd1*-KO mice was not observed. This was unexpected, as 10% of glycogen degraded in the liver passes through the lysosomal pathway [55] and the loss of STBD1 should result in an appreciable accumulation of glycogen in the liver. One explanation for the lack of this glycogen accumulation is the compensation of STBD1-mediated glycogen translocation into lysosomes by cytoplasmic glycogen degradation. Alternative mechanisms of glycogen translocation are also possible, as the loss of STBD1 in a mouse model of lysosomal glycogen overload (acid alpha-glucosidase-KO mice) did not completely antagonize lysosomal glycogen accumulation [43]. Hence, we conclude that reduction of STBD1 in CCF may only contribute to glycogen accumulation in combination with yet unidentified factors that favor lysosomal glycogen degradation. As large glycogen granulae are preferentially degraded via the lysosomal pathway [55], it would be of interest to determine whether glycogen granulae in CCF are of a larger size than in normal liver tissue. An alternative interpretation of the reduction of STBD1 in CCF could be a rerouting of carbohydrate use. Reducing the utilization of glycogen for lysosomal degradation and glucose export would result in increased intracellular glucose availability. Indeed, cancer cells have been shown to route glucose through glycogen and this glucose was able to support the pentose phosphate pathway more optimally [56,57].

### 3.3. USP28

With regard to hepatocellular carcinogenesis, USP28 is of particular interest. Functionally, USP28 is a deubiquitination enzyme, catalyzing the deubiquitination of target proteins [58,59], thereby counteracting ubiquitin-dependent proteasomal protein degradation. Known target proteins of USP28 are 53BP1 [44,45,46] and claspin [59], MYC [60], LSD1 [61], histone H2A [62], and HIF-1alpha [63]. In particular, lack of USP28 results in an earlier onset and greater tumor burden in a mouse model of chemically (diethylnitrosamine (DEN)) induced hepatocellular carcinoma [25]. The same study also reports that USP28 expression is reduced in patients with hepatocellular carcinoma when comparing carcinoma with control liver tissue from the same patient [25], in line with the data from the current study. Functions of USP28 in different cancer cell types reveal a variety of cancer-relevant effects of USP28: it elicits stem-cell-like characteristics through LSD1-stabilization [61], it acts on p53 and GATA4, affecting cellular senescence [64], it impacts on cell proliferation through deubiquitination of histone H2A [62], and it sensitizes cells to DNA-damage via its interactions with 53BP1 and claspin [59]. Finally, USP28 has been targeted in several studies for the treatment of different cancers, such as non-small cell lung cancer, breast cancer, intestinal cancers, gliomas, and bladder cancer [65]. Our data (reduced *USP28* mRNA in CCF, validated at the protein level by immunohistochemistry) reveal changes in USP28 expression in small human hepatocellular foci, providing further evidence for CCF being very early lesions in hepatocellular carcinogenesis or pre-neoplastic lesions, respectively. As we did not find any evidence that a lack of USP28 would induce glycogen overload in DEN-induced hepatocellular carcinoma, we propose that the reduced expression of USP28 preferentially affects the reported p53/senescence pathway axis to promote tumorigenesis and cancerogenesis in CCF rather than having an additional function in regulating glycogen metabolism. To our knowledge, the alteration of USP28 expression in early clear cell lesions is the first of its kind and it will be interesting to investigate mechanisms that trigger the reduced USP28 expression in CCF. Our results of reduced *USP28* mRNA expression (microarrays) and reduced USP28 protein expression (IHC) suggest that the differential regulation is mediated at the transcript level. What causes the differential USP28-expression and what effects it has on CCF progression to tumors will be the focus of future studies.

## 4. Conclusions

With our omics approach, we identified several new genes/proteins that show differential expression in CCF. Initial functional studies indicate that reduced expression of STBD1 may not have a direct effect on glycogen levels despite its role in glycogen degradation. Most likely, other factors also contribute and need to be identified in dedicated studies.

Furthermore, the reduced expression of USP28, a gene known to affect onset and progression of liver and breast cancer [25], is likely to mediate glycogen-independent oncogenic functions. Our work is the first to show that the expression of USP28 is already decreased in CCF, underscoring their pre-neoplastic character.

## 5. Materials and Methods

### 5.1. Human Liver Specimens

Liver samples originated from a former cohort [20] without signs of liver cirrhosis, obtained from human liver resections taken during surgery. Specimens were from patients (age ranging from 42 to 77 years) with liver metastases of tumors of different origin (two colon carcinomas, two neuroendocrine tumors, one gastrointestinal stromal tumor) or with cholangiocellular carcinoma (n = 4). Tissue samples (1.5 × 1.5 × 0.5 cm) were collected and frozen in liquid nitrogen-cooled isopentane and stored at −80 °C until cryosectioning. Experiments were reviewed and permitted by the ethical committee of the Universitaetsmedizin Greifswald (No. BB 67/10).

### 5.2. Cryosectioning 

Cryosections were made at −16 to −20 °C using a Cryostar NX (Thermo Scientific, Waltham, MA, USA) disinfected with Leica Cryofect Disinfectant Spray (Leica, Wetzlar, Germany) before use. All equipment was cleaned with RNase AWAY (Molecular Bio Products, San Diego, CA, USA) and glassware was additionally rinsed with RNase-free water (Aqua B. Braun, Ecotainer, B. Braun Melsungen AG, Melsungen, Germany) and a new blade was used for every sample to reduce risks of cross-contamination. Samples were attached to an object-holder with 0.9% sodium chloride solution (NaCl 0.9%; B. Braun Melsungen AG, Melsungen, Germany). Specimens with clear cell foci were identified by H&E staining of 8 µm sections and validated by PAS reaction. In regions with CCF, two to four consecutive cryosections with a thickness of 12 µm were put on membrane-slides (Leica Frame Slides, Nuclease and human nucleic acid free, PET-Membrane, 1.4 µm, Leica, Wetzlar, Germany) and incubated for one minute in 70% ethanol at −16 °C. Membrane slides were collected in a box, vacuum-sealed (Severin FS 3602, Severin Elektrogeräte GmbH, Sundern, Germany), and stored at −80 °C until laser micro-dissection.

### 5.3. Laser Micro-Dissection

CCF and control samples were isolated by laser micro-dissection using a Leica LMD 6500 System (Leica Microsystems, Wetzlar, Germany) wiped with RNase AWAY (Molecular Bio Products, San Diego, CA, USA). Sample boxes were thawed for 30 min on ice before staining the cryosections according to a modified H&E-staining protocol. Briefly: sections were incubated in DEPC-water (0.1% diethyl-pyrocarbonate, Sigma-Aldrich, St. Louis, MO, USA) for ten sec, stained with hemalaun for 50 sec, and washed for 10 sec with DEPC-water before staining with eosin for 10 sec. Slides were then incubated in 90% ethanol for 30 sec.

Material from the same patient and same sample type was collected in one tube and stored on dry ice during collection. For storage, 800 µL TRIzol Reagent (Life Technologies, Carlsbad, CA, USA) was added and samples were stored at −80 °C.

For microarray analysis, nine (N = 9), and for proteomic analysis, seven, sample pairs (N = 7) were of suitable quality. 

### 5.4. RNA and Protein Isolation

Samples (on average 91 (CCF) and 96 (control) dissected tissue pieces per patient) were homogenized in liquid nitrogen, pre-cooled in 4 mL PTFE-vials with one 8 mm stainless-steel bead using a Micro-Dismembranator (Sartorius AG, Göttingen) at 2600 rotations per minute (RPM) for two min. The homogenate was transferred into a 1.7 mL centrifuge tube (Sorenson Bioscience Inc., Murray, UT, USA) and the homogenization vials were flushed with another 200 μL TRIzol. Samples stored on dry ice were thawed at room temperature for ten min, then centrifuged at 4 °C and 12,000× *g* for ten min (Heraeus Fresco 17 Centrifuge Refrigerated, Thermo Scientific, Waltham, MA, USA) to remove crude debris.

RNA and proteins were isolated using TRIzol extraction according to the manufacturer’s protocol with the following changes. RNA was precipitated with isopropanol overnight at −20 °C, RNA was washed twice with 70% ethanol, and pellets were resuspended by incubation on water ice for three h, followed by 30 min of incubation at room temperature.

Protein pellets were solubilized according to the manufacturer’s protocols; for smaller pellets, 50 µL 1% SDS (Sodium Dodecyl Sulfate, Bio-Rad Laboratories Inc., Hercules, CA, USA) was used.

RNA was quantified using a Nanodrop 8000 (Thermo Scientific, Waltham, MA, USA) and RNA quality was assessed using a Bioanalyzer (Agilent 2100 Bioanalyzer, Agilent Technologies, Santa Clara, CA, USA) (Supplementary Table S1).

### 5.5. Microarray Analysis

Processing of purified RNA for microarray analysis and the microarray analysis were carried out at OakLabs (Hennigsdorf, Germany) according to their standard procedures. Briefly, RNA concentrations were between 17 and 66 ng/µL in volumes of 30 or 40 µL H_2_O with integrity numbers (RIN, Bioanalyzer, Agilent Technologies, USA) between 6.3 and 7.9 (Supplementary Table S1). RNA was labeled using the Low-Input QuickAmp Labeling Kit (Agilent Technologies, USA) and cRNA was hybridized with ArrayXS Human (OakLabs, Germany) at 65 °C for 17 h using the Agilent Gene Expression Hybridization Kit (Agilent Technologies, USA), washed once with Agilent Gene Expression Wash Buffer 1 for one minute at room temperature, followed by a second wash with preheated (37 °C) Gene Expression Wash Buffer 2 for one minute.

Microarrays were scanned with a SureScan Microarray Scanner (Agilent Technologies, USA) and Agilent´s Feature Extraction software was used to detect features. Signals from control probes were removed and means of signals from replicate probes and of signals from all probes of a target were determined before normalization of the background subtracted signals. Data from all samples was quantile normalized using ranked mean quartiles [66]. Normalized data was statistically analyzed by paired analysis of variance (ANOVA) (clear cell foci vs. control samples from the same patient).

### 5.6. Gel Electrophoresis and Silver Staining

Protein concentrations were determined by Bradford assay (Quick Start Bradford Protein Assay, Bio-Rad Laboratories Inc., Hercules, CA, USA) (average concentration: 1.24 µg/µL +/− 0.44). To assess protein integrity, 13.7–20 µg total protein per sample was resolved under reducing conditions on Novex NuPAGE gels (4–12% bis-tris protein gels) using LDS sample buffer and MES running buffer according to the manufacturer’s protocol (Life Technologies, Carlsbad, USA). Gels were stained using the Silver Staining Plus Kit and visualized on the ChemiDoc XRS+ system (Bio-Rad Laboratories Inc., Hercules, CA, USA) according to the manufacturer’s protocol.

### 5.7. Proteomics Sample Preparation and LC-MS/MS

Sample preparation and mass-spectrometric analysis was carried out by the proteomic facility of Porto Conte Ricerche (Alghero, Italy). Protein extracts were subjected to on-filter reduction, alkylation, and trypsin digestion according to the filter-aided sample preparation (FASP) protocol, with slight modifications. Briefly, protein extracts were diluted in 8 M urea in Tris-HCl 100 mM pH 8.8, and buffer was exchanged using Microcon Ultracel YM-10 filtration devices (Millipore, Billerica, MA, USA). Proteins were reduced in 10 mM dithiothreitiol (DTT) for 30 min, alkylated in 50 mM iodoacetamide for 20 min, washed five times (3× in 8 M urea and 2× in ammonium bicarbonate), before trypsin digestion on the filter (1:100 enzyme-to-protein ratio) at 37 °C overnight. Peptides were collected by centrifugation, followed by an additional wash with an elution solution (70% acetonitrile plus 1% formic acid). Finally, the peptide mixture was dried, and reconstituted in 0.2% formic acid to an approximate final concentration of 1 µg/µL. Peptide mixture concentration was estimated by measuring absorbance at 280 nm with a NanoDrop 2000 spectrophotometer (Thermo Scientific, San Jose, CA, USA) and a standard curve made from MassPREP *E. Coli* Digest Standard (Waters, Milford, MA, USA).

Liquid Chromatography with tandem mass spectrometry (LC-MS/MS) analyses were carried out using a Q Exactive mass spectrometer (Thermo Scientific) interfaced with an UltiMate 3000 RSLCnano LC system (Thermo Scientific). After loading, peptide mixtures (4 μg per run) were concentrated and desalted on a trapping pre-column (Acclaim PepMap C18, 75 μm × 2 cm nanoViper, 3 μm, 100 Å, Thermo Scientific), using 0.2% formic acid at a flow rate of 5 μL/min. The peptide separation was performed at 35 °C using a C18 column (EASY-Spray column, 15 cm × 75 μm ID, PepMap C18, 3 μm, Thermo Scientific) at a flow rate of 300 nL/min, using a 485 min gradient from 1 to 50% eluent B (0.2% formic acid in 95% acetonitrile) in eluent A (0.2% formic acid in 5% acetonitrile). MS data were acquired using a data-dependent top10 method dynamically choosing the most abundant precursor ions from the survey scan, under direct control of the Xcalibur software (version 1.0.2.65 SP2), where a full-scan spectrum (from 300 to 1700 m/z) was followed by tandem mass spectra (MS/MS). The instrument was operated in positive mode with a spray voltage of 1.8 kV and a capillary temperature of 275 °C. Survey and MS/MS scans were performed in the Orbitrap with resolution of 70,000 and 17,500 at 200 m/z, respectively. The automatic gain control was set to 1,000,000 ions and the lock mass option was enabled on a protonated polydimethylcyclosiloxane background ion as internal recalibration for accurate mass measurements. The dynamic exclusion was set to 30 sec. Higher Energy Collisional Dissociation (HCD), performed at the far side of the C-trap, was used as the fragmentation method, by applying a 25 eV value for normalized collision energy, and an isolation width of m/z 2.0. Nitrogen was used as the collision gas.

Peptide identification was performed using Proteome Discoverer (version 1.4; Thermo Scientific) using Sequest-HT as a search engine for protein identification, according to the following criteria: Database UniprotKB, taxonomy human (release 2014_10); Precursor mass tolerance: 10 ppm; Fragment mass tolerance: 0.02 Da; Static modification: cysteine carbamidomethylation; Dynamic modification: methionine oxidation, and Percolator for peptide validation (false discovery rate (FDR) < 1% based on peptide q-value). Results were filtered in order to keep only rank 1 peptides, and protein grouping was allowed according to the maximum parsimony principle.

Protein abundance was expressed by means of the normalized spectral abundance factor (NSAF). NSAF was calculated as follows: NSAF = SAF_i_/N, where the subscript *i* denotes a protein identity and N is the total number of proteins, while SAF is a protein spectral abundance factor that is defined as the protein spectral counts divided by its length (number of residues or molecular weight). In this approach, the spectral counts of each protein were divided by its length and normalized to the average number of spectral counts in a given analysis. In order to eliminate discontinuity due to Spectral counts = 0, a correction factor, set to 2, was used. The NSAF log ratio (RNSAF) was calculated according to the following formula: RNSAF = log2(NSAF1/NSAF2), where RNSAF is the log2 ratio of the abundance of a protein in sample groups 1 (clear cell foci, NSAF1) and 2 (control, NSAF2).

Proteins showing RNSAF > 0.5 or < −0.5 were considered as differentially abundant between groups. A two-tailed *t*-test was applied, using in-house software, in order to evaluate the statistical significance of differences between groups. No correction for multiple testing was applied. To address the potentially higher rate of false-positive hits, the most interesting candidates were validated by immunohistochemistry.

### 5.8. Cluster Analysis and Plotting of Omics Data

Cluster analysis of proteomic and microarray data was done using Perseus Software (version 1.6.0.7,). Microarray data: quartile normed data was filtered by intensity (signal ≥ 10, in 8 out of 9 samples within one group; groups: normal and CCF). Settings for hierarchical clustering: Distance—Euclidean; Linkage-Average (process with k-means, number of clusters: 300, maximal number of iterations: 10, number of restarts: 1).

Data plotting was done using R (3.5.2) with the following packages: ggplot2, ggrepel, and gridExtra.

### 5.9. Immunohistochemistry and Histochemistry

Histochemistry of formaldehyde-fixed and paraffin-embedded specimen was performed as previously described [14].

Immunohistochemistry was carried out on formaldehyde-fixed and paraffin-embedded specimens according to standard immunohistochemical protocols for de-paraffination and embedding. Endogenous peroxidase was blocked with Novocastra^TM^ Peroxidase Block (#RE7101, Leica Biosystems) for 15 min at room temperature and sections were blocked with Universal Block (Dako) for 20 min. Primary antibodies and unmasking techniques are listed in Supplementary Table S2. LSAB2 System-HRP (#K0675, Dako) and Liquid DAB+ Substrate Chromogen System (#K3468, Dako) were used for signal amplification and staining.

### 5.10. Mouse Models

*Stbd1*-KO mice [43] and the respective control animals were bred and housed at Duke University, USA. All animal procedures were done in accordance with Duke University Institutional Animal Care and Use Committee-approved guidelines.

Sections of *Usp28*-KO mice were prepared as described [25].

### 5.11. Glycogen Quantification in Mouse Liver

Glycogen was quantified in mouse liver as described previously [67].

## Figures and Tables

**Table 1:** Genes significantly changed between CCF (clear cell foci) and unaltered liver tissue (at least 2-fold up/down, CCF vs. control; *p*-value ≤ 0.05).

Target Name	Gene Symbol	Gene Product	log2(FC)	*p*-Value	Function
lnc-C14orf23-6:1 lnc-FOXG1-6:17(LNCIPEDIA v5.2)	lnc-C14orf23-6	lnc-FOXG1-6, (ENSG00000257120.1; CTD-2503I6.1; OTTHUMG00000170489.1; AL356756.1)	1.25	0.0439	lncRNA; antisense to PRKD1; +strand; 2 exons; 551 bp; in stringent set
NR_027433.1 LINC01124:6 (LNCIPEDIA v5.2)	LINC01124	long intergenic non-protein coding RNA 1124	1.08	0.0047	lncRNA; bidirectional; -strand; 1 exon; 2129 bp; in stringent set
lnc-FUT8-2:12 LINC02290:27 (LNCIPEDIA v5.2)	lnc-FUT8-2 LINC02290 (LNCIPEDIA v5.2)	XLOC_010856; linc-GPHN-2	1.04	0.0051	lncRNA; intergenic; +strand; 4 exons; 463 bp; not in stringent set
NM_032413.3	C15orf48	chromosome 15 open reading frame 48	−1.03	0.0496	no reported function
NM_001145434.1	ZNF880	zinc finger protein 880	−1.05	0.0269	no reported function
NM_001301029.1	USP28	Ubiquitin carboxyl-terminal hydrolase 28	−1.09	0.0320	protein deubiquitination; c-Myc stabilization and hepatocarcinogenesis [24,25]
lnc-C15orf41-2:1 (LNCIPEDIA V5.2)	lnc-C15orf41-2	lincRNA	−1.14	0.0217	lncRNA; intergenic; +strand; 2 exons; 393 bp; in stringent set
NM_173538.2	CNBD1	cyclic nucleotide binding domain containing 1	−1.16	0.0378	
NM_032553.1	GPR174	G protein-coupled receptor 174	−1.19	0.0250	lysophosphatidylserine receptor [26,27]; SNPs are risk factors for Graves’ disease [28]; autoimmune Addison´s disease [29];
NM_007210.3	GALNT6	polypeptide N-acetylgalactosaminyltransferase 6	−1.20	0.0108	marker for early tumorigenesis in breast cancer [30]; suppressor of colorectal cancer progression 30662801; enhancer of aggression of ovarian cancer cells [31]; protection from apoptosis under stress conditions [32]; promotion of tumorigenicity and metastasis in breast cancer [33]; growth suppression in pancreatic cancer [34]
NM_001256476.1	WDR92	monad/WD repeat domain 92	−1.22	0.0198	part of the R2TP/prefoldin-like complex [35]; inhibits breast cancer cell invasion [36]; TNFalpha/cycloheximide-mediated apoptosis [37]
NM_005185.3	CALML3	calmodulin-like 3	−1.30	0.0069	calcium homeostasis (by similarity); suppression of gastric cancer progression by secreted CALML3 after metformin treatment [38]
NM_001281739.1	FHOD3	Formin Homology 2 Domain Containing 3	−1.46	0.0350	actin binding 24914801; cancer cell invasiveness [39]
NM_006235.2	POU2AF1	POU class 2 associating factor 1	−1.50	0.0185	B cell maturation 17621271

**Table 2:** Proteins with statistically significant differential expression in CCF vs. control tissue (Student’s *t*-test, N = 7).

Accession	Description (Gene Symbol)	log2 Ratio (CCF/Control)	*t*-Test *p*-Value
**higher expression in CCF**			
Q15286	Ras-related protein Rab-35 (*RAB35*)	0.9087	0.0447
Q6IQ22	Ras-related protein Rab-12 (*RAB12*)	0.7768	0.0439
Q03013	Glutathione S-transferase Mu 4 (GSTM4)	0.6028	0.0066
**lower expression in *CCF***			
Q12797	Aspartyl/asparaginyl beta-hydroxylase (*ASPH*)	−0.6097	0.0254
Q15008	26S proteasome non-ATPase regulatory subunit 6 (*PSMD6*)	−0.6155	0.0121
P84098	60S ribosomal protein L19 (*RPL19*)	−0.6163	0.0086
P61247	40S ribosomal protein S3a (*RPS3A*)	−0.6171	0.0474
Q13451	Peptidyl-prolyl cis-trans isomerase FKBP5 (*FKBP*)	−0.6322	0.0489
Q9UNF0	Protein kinase C and casein kinase substrate in neurons protein 2 (*PACSIN2*)	−0.6591	0.0327
O95810	Serum deprivation-response protein (*SDPR*)	−0.6682	0.0357
Q16836	Hydroxyacyl-coenzyme A dehydrogenase, mitochondrial (*HADH*)	−0.6846	0.0146
O75821	Eukaryotic translation initiation factor 3 subunit G (*EIF3G*)	−0.7156	0.0333
Q9BVK6	Transmembrane emp24 domain-containing protein 9 (*TMED9*)	−0.7615	0.021
P63208	S-phase kinase-associated protein 1 (*SKP1*)	−0.7715	0.0282
Q15436	Protein transport protein Sec23A (*SEC23A*)	−0.7936	0.0441
Q16629	Serine/arginine-rich splicing factor 7 (*SRSF7*)	−0.8146	0.026
Q95604	HLA class I histocompatibility antigen, Cw-17 alpha chain (*HLA-C*)	−0.8195	0.0429
O75436	Vacuolar protein sorting-associated protein 26A (*VPS26A*)	−0.878	0.0217
Q9NRV9	Heme-binding protein 1 (*HEBP1*)	−0.9163	0.034
O95210	Starch-binding domain-containing protein 1 (*STBD1*)	−1.1576	0.0035
P61604	10 kDa heat shock protein, mitochondrial (*HSPE1*)	−1.1642	0.0326
O43169	Cytochrome b5 type B (*CYB5B*)	−1.211	0.0157

**Table 3:** Validation of protein expression as predicted by high-throughput analysis (see Table 1 and Table 2 for fold changes) in CCF by immunohistochemical analysis. Arrows indicate higher (↑), lower (↓), and unchanged (←→) expression in CCF compared to unaltered liver tissue. Numbers in the immunohistochemistry column denote the number of individual patient samples analyzed.

	CCF vs. Control	
Protein	High-Throughput Analysis	Immunohistochemistry
CYB5B	↓ (protein)	↑ 0 ←→ 2 ↓ 9
HSPE1	↓ (protein)	↑ 0 ←→ 5 ↓ 1 ↓ 4
monad/WDR92	↓ (mRNA)	↑ 0 ←→ 2 ↓ 9
RAB12	↑ (protein)	↑ 0 ←→ 7 ↓ 3
RAB35	↑ (protein)	↑ 0 ←→ 7 ↓ 3
STBD1	↓ (protein)	↑ 0 ←→ 0 ↓ 11
USP28	↓ (mRNA)	↑ 0 ←→ 1 ↓ 6

## Supplementary Materials

The following are available online, Figure S1: Number of transcripts per sample, Figure S2: Histograms of log2 transformed RNA expression, Figure S3: Number of proteins identified per sample, Figure S4: Histograms of log2 transformed protein expression, Figure S5: Immunohistochemistry of negative controls, Table S1: RNA concentration and quality, Table S2: Antibodies used in this study.

## References

[1] Franco L.M., Krishnamurthy V., Bali D., Weinstein D.A., Arn P., Clary B., Boney A., Sullivan J., Frush D.P., Chen Y.-T. (2005). Hepatocellular carcinoma in glycogen storage disease type Ia: A case series. J. Inherit. Metab. Dis..

[2] Marengo A., Rosso C., Bugianesi E. (2016). Liver Cancer: Connections with Obesity, Fatty Liver, and Cirrhosis. Annu. Rev. Med..

[3] Davila J.A., Morgan R.O., Shaib Y., McGlynn K.A., El-Serag H.B. (2005). Diabetes increases the risk of hepatocellular carcinoma in the United States: A population based case control study. Gut.

[4] Vigneri R., Goldfine I.D., Frittitta L. (2016). Insulin, insulin receptors, and cancer. J. Endocrinol. Invest..

[5] Libbrecht L., Desmet V., Roskams T. (2005). Preneoplastic lesions in human hepatocarcinogenesis. Liver Int..

[6] Evert M., Dombrowski F. (2008). Hepatocellular carcinoma in the non-cirrhotic liver. Pathologe.

[7] Bannasch P., Ribback S., Su Q., Mayer D. (2017). Clear cell hepatocellular carcinoma: Origin, metabolic traits and fate of glycogenotic clear and ground glass cells. HBPD INT.

[8] Bannasch P. (1996). Pathogenesis of hepatocellular carcinoma: Sequential cellular, molecular, and metabolic changes. Prog Liver Dis.

[9] Bannasch P., Mayer D., Hacker H.J. (1980). Hepatocellular glycogenosis and hepatocarcinogenesis. Biochim. Biophys. Acta.

[10] Williams G.M. (1980). The pathogenesis of rat liver cancer caused by chemical carcinogens. Biochim. Biophys. Acta.

[11] Pitot H.C. (1990). Altered hepatic foci: Their role in murine hepatocarcinogenesis. Annu. Rev. Pharmacol. Toxicol..

[12] Dombrowski F., Filsinger E., Bannasch P., Pfeifer U. (1996). Altered liver acini induced in diabetic rats by portal vein islet isografts resemble preneoplastic hepatic foci in their enzymic pattern. Am. J. Pathol..

[13] Dombrowski F., Mathieu C., Evert M. (2006). Hepatocellular neoplasms induced by low-number pancreatic islet transplants in autoimmune diabetic BB/Pfd rats. Cancer Res..

[14] Dombrowski F., Bannasch P., Pfeifer U. (1997). Hepatocellular neoplasms induced by low-number pancreatic islet transplants in streptozotocin diabetic rats. Am. J. Pathol..

[15] Dombrowski F., Jost C.M., Manekeller S., Evert M. (2005). Cocarcinogenic effects of islet hormones and N-nitrosomorpholine in hepatocarcinogenesis after intrahepatic transplantation of pancreatic islets in streptozotocin-diabetic rats. Cancer Res..

[16] Ribback S., Cigliano A., Kroeger N., Pilo M.G., Terracciano L., Burchardt M., Bannasch P., Calvisi D.F., Dombrowski F. (2015). PI3K/AKT/mTOR pathway plays a major pathogenetic role in glycogen accumulation and tumor development in renal distal tubules of rats and men. Oncotarget.

[17] Evert M., Calvisi D.F., Evert K., De Murtas V., Gasparetti G., Mattu S., Destefanis G., Ladu S., Zimmermann A., Delogu S. (2012). V-AKT murine thymoma viral oncogene homolog/mammalian target of rapamycin activation induces a module of metabolic changes contributing to growth in insulin-induced hepatocarcinogenesis. Hepatology.

[18] Calvisi D.F., Wang C., Ho C., Ladu S., Lee S.A., Mattu S., Destefanis G., Delogu S., Zimmermann A., Ericsson J. (2011). Increased lipogenesis, induced by AKT-mTORC1-RPS6 signaling, promotes development of human hepatocellular carcinoma. Gastroenterology.

[19] Calvisi D.F., Ladu S., Gorden A., Farina M., Conner E.A., Lee J.-S., Factor V.M., Thorgeirsson S.S. (2006). Ubiquitous activation of Ras and Jak/Stat pathways in human HCC. Gastroenterology.

[20] Ribback S., Calvisi D.F., Cigliano A., Sailer V., Peters M., Rausch J., Heidecke C.-D., Birth M., Dombrowski F. (2013). Molecular and metabolic changes in human liver clear cell foci resemble the alterations occurring in rat hepatocarcinogenesis. J. Hepatol..

[21] Ribback S., Sonke J., Lohr A., Frohme J., Peters K., Holm J., Peters M., Cigliano A., Calvisi D.F., Dombrowski F. (2017). Hepatocellular glycogenotic foci after combined intraportal pancreatic islet transplantation and knockout of the carbohydrate responsive element binding protein in diabetic mice. Oncotarget.

[22] Li L., Che L., Tharp K.M., Park H.-M., Pilo M.G., Cao D., Cigliano A., Latte G., Xu Z., Ribback S. (2016). Differential requirement for de novo lipogenesis in cholangiocarcinoma and hepatocellular carcinoma of mice and humans. Hepatology.

[23] Che L., Pilo M.G., Cigliano A., Latte G., Simile M.M., Ribback S., Dombrowski F., Evert M., Chen X., Calvisi D.F. (2017). Oncogene dependent requirement of fatty acid synthase in hepatocellular carcinoma. Cell Cycle.

[24] Han H., Sun D., Li W., Shen H., Zhu Y., Li C., Chen Y., Lu L., Li W., Zhang J. (2013). A c-Myc-MicroRNA functional feedback loop affects hepatocarcinogenesis. Hepatology.

[25] Richter K., Paakkola T., Mennerich D., Kubaichuk K., Konzack A., Kippari H.A., Kozlova N., Koivunen P., Haapasaari K.-M., Jukkola-Vuorinen A. (2018). USP28 Deficiency Promotes Breast and Liver Carcinogenesis as well as Tumor Angiogenesis in a HIF-independent Manner. Mol. Cancer Res..

[26] Uwamizu A., Inoue A., Suzuki K., Okudaira M., Shuto A., Shinjo Y., Ishiguro J., Makide K., Ikubo M., Nakamura S. (2015). Lysophosphatidylserine analogues differentially activate three LysoPS receptors. J. Biochem..

[27] Makide K., Uwamizu A., Shinjo Y., Ishiguro J., Okutani M., Inoue A., Aoki J. (2014). Novel lysophosphoplipid receptors: Their structure and function. J. Lipid Res..

[28] Chu X., Shen M., Xie F., Miao X.-J., Shou W.-H., Liu L., Yang P.-P., Bai Y.-N., Zhang K.-Y., Yang L. (2013). An X chromosome-wide association analysis identifies variants in GPR174 as a risk factor for Graves’ disease. J. Med. Genet..

[29] Napier C., Mitchell A.L., Gan E., Wilson I., Pearce S.H.S. (2015). Role of the X-linked gene GPR174 in autoimmune Addison’s disease. J. Clin. Endocrinol. Metab..

[30] Andergassen U., Liesche F., Kölbl A.C., Ilmer M., Hutter S., Friese K., Jeschke U. (2015). Glycosyltransferases as Markers for Early Tumorigenesis. Biomed Res Int.

[31] Lin T.-C., Chen S.-T., Huang M.-C., Huang J., Hsu C.-L., Juan H.-F., Lin H.-H., Chen C.-H. (2017). GALNT6 expression enhances aggressive phenotypes of ovarian cancer cells by regulating EGFR activity. Oncotarget.

[32] Lin J., Chung S., Ueda K., Matsuda K., Nakamura Y., Park J.-H. (2017). GALNT6 Stabilizes GRP78 Protein by O-glycosylation and Enhances its Activity to Suppress Apoptosis Under Stress Condition. Neoplasia.

[33] Mao Y., Zhang Y., Fan S., Chen L., Tang L., Chen X., Lyu J. (2019). GALNT6 Promotes Tumorigenicity and Metastasis of Breast Cancer Cell via β-catenin/MUC1-C Signaling Pathway. Int. J. Biol. Sci..

[34] Tarhan Y.E., Kato T., Jang M., Haga Y., Ueda K., Nakamura Y., Park J.-H. (2016). Morphological Changes, Cadherin Switching, and Growth Suppression in Pancreatic Cancer by GALNT6 Knockdown. Neoplasia.

[35] von Morgen P., Hořejší Z., Macurek L. (2015). Substrate recognition and function of the R2TP complex in response to cellular stress. Front. Genet..

[36] Saeki M., Egusa H., Kamano Y., Kakihara Y., Houry W.A., Yatani H., Noguchi S., Kamisaki Y. (2013). Exosome-bound WD repeat protein Monad inhibits breast cancer cell invasion by degrading amphiregulin mRNA. PLoS ONE.

[37] Saeki M., Irie Y., Ni L., Yoshida M., Itsuki Y., Kamisaki Y. (2006). Monad, a WD40 repeat protein, promotes apoptosis induced by TNF-alpha. Biochem. Biophys. Res. Commun..

[38] Chen G., Yu C., Tang Z., Liu S., An F., Zhu J., Wu Q., Cao J., Zhan Q., Zhang S. (2019). Metformin suppresses gastric cancer progression through calmodulin-like protein 3 secreted from tumor-associated fibroblasts. Oncol. Rep..

[39] Paul N.R., Allen J.L., Chapman A., Morlan-Mairal M., Zindy E., Jacquemet G., Fernandez del Ama L., Ferizovic N., Green D.M., Howe J.D. (2015). α5β1 integrin recycling promotes Arp2/3-independent cancer cell invasion via the formin FHOD3. J. Cell Biol..

[40] Zhao C., Inoue J., Imoto I., Otsuki T., Iida S., Ueda R., Inazawa J. (2008). POU2AF1, an amplification target at 11q23, promotes growth of multiple myeloma cells by directly regulating expression of a B-cell maturation factor, TNFRSF17. Oncogene.

[41] Jiang S., Heller B., Tagliabracci V.S., Zhai L., Irimia J.M., Depaoli-Roach A.A., Wells C.D., Skurat A.V., Roach P.J. (2010). Starch binding domain-containing protein 1/genethonin 1 is a novel participant in glycogen metabolism. J. Biol. Chem..

[42] Jiang S., Wells C.D., Roach P.J. (2011). Starch-binding domain-containing protein 1 (Stbd1) and glycogen metabolism: Identification of the Atg8 family interacting motif (AIM) in Stbd1 required for interaction with GABARAPL1. Biochem. Biophys. Res. Commun..

[43] Sun T., Yi H., Yang C., Kishnani P.S., Sun B. (2016). Starch Binding Domain-containing Protein 1 Plays a Dominant Role in Glycogen Transport to Lysosomes in Liver. J. Biol. Chem..

[44] Lambrus B.G., Daggubati V., Uetake Y., Scott P.M., Clutario K.M., Sluder G., Holland A.J. (2016). A USP28-53BP1-p53-p21 signaling axis arrests growth after centrosome loss or prolonged mitosis. J. Cell Biol..

[45] Fong C.S., Mazo G., Das T., Goodman J., Kim M., O’Rourke B.P., Izquierdo D., Tsou M.-F.B. (2016). 53BP1 and USP28 mediate p53-dependent cell cycle arrest in response to centrosome loss and prolonged mitosis. Elife.

[46] Meitinger F., Anzola J.V., Kaulich M., Richardson A., Stender J.D., Benner C., Glass C.K., Dowdy S.F., Desai A., Shiau A.K. (2016). 53BP1 and USP28 mediate p53 activation and G1 arrest after centrosome loss or extended mitotic duration. J. Cell Biol..

[47] Vogel C., Marcotte E.M. (2012). Insights into the regulation of protein abundance from proteomic and transcriptomic analyses. Nat. Rev. Genet..

[48] Latgé G., Poulet C., Bours V., Josse C., Jerusalem G. (2018). Natural Antisense Transcripts: Molecular Mechanisms and Implications in Breast Cancers. Int J Mol Sci.

[49] Youssef I., Ricort J.-M. (2019). Deciphering the role of protein kinase D1 (PKD1) in cellular proliferation. Mol. Cancer Res..

[50] Uhlen M., Zhang C., Lee S., Sjöstedt E., Fagerberg L., Bidkhori G., Benfeitas R., Arif M., Liu Z., Edfors F. (2017). A pathology atlas of the human cancer transcriptome. Science.

[51] Thul P.J., Åkesson L., Wiking M., Mahdessian D., Geladaki A., Ait Blal H., Alm T., Asplund A., Björk L., Breckels L.M. (2017). A subcellular map of the human proteome. Science.

[52] Uhlen M., Fagerberg L., Hallström B.M., Lindskog C., Oksvold P., Mardinoglu A., Sivertsson Å., Kampf C., Sjöstedt E., Asplund A. (2015). Proteomics. Tissue-based map of the human proteome. Science.

[53] Bouju S., Lignon M.F., Piétu G., Le Cunff M., Léger J.J., Auffray C., Dechesne C.A. (1998). Molecular cloning and functional expression of a novel human gene encoding two 41-43 kDa skeletal muscle internal membrane proteins. Biochem. J..

[54] Janeček Š. (2002). A motif of a microbial starch-binding domain found in human genethonin. Bioinformatics.

[55] Prats C., Graham T.E., Shearer J. (2018). The dynamic life of the glycogen granule. J. Biol. Chem..

[56] Klimek F., Mayer D., Bannasch P. (1984). Biochemical microanalysis of glycogen content and glucose-6-phosphate dehydrogenase activity in focal lesions of the rat liver induced by N-nitrosomorpholine. Carcinogenesis.

[57] Favaro E., Bensaad K., Chong M.G., Tennant D.A., Ferguson D.J.P., Snell C., Steers G., Turley H., Li J.-L., Günther U.L. (2012). Glucose Utilization via Glycogen Phosphorylase Sustains Proliferation and Prevents Premature Senescence in Cancer Cells. Cell Metabolism.

[58] Valero R., Bayés M., Francisca Sánchez-Font M., González-Angulo O., Gonzàlez-Duarte R., Marfany G. (2001). Characterization of alternatively spliced products and tissue-specific isoforms of USP28 and USP25. Genome Biol..

[59] Zhang D., Zaugg K., Mak T.W., Elledge S.J. (2006). A role for the deubiquitinating enzyme USP28 in control of the DNA-damage response. Cell.

[60] Popov N., Wanzel M., Madiredjo M., Zhang D., Beijersbergen R., Bernards R., Moll R., Elledge S.J., Eilers M. (2007). The ubiquitin-specific protease USP28 is required for MYC stability. Nat Cell Biol.

[61] Wu Y., Wang Y., Yang X.H., Kang T., Zhao Y., Wang C., Evers B.M., Zhou B.P. (2013). The deubiquitinase USP28 stabilizes LSD1 and confers stem-cell-like traits to breast cancer cells. Cell Rep.

[62] Li F., Han H., Sun Q., Liu K., Lin N., Xu C., Zhao Z., Zhao W. (2019). USP28 regulates deubiquitination of histone H2A and cell proliferation. Exp. Cell Res..

[63] Flügel D., Görlach A., Kietzmann T. (2012). GSK-3β regulates cell growth, migration, and angiogenesis via Fbw7 and USP28-dependent degradation of HIF-1α. Blood.

[64] Mazzucco A.E., Smogorzewska A., Kang C., Luo J., Schlabach M.R., Xu Q., Patel R., Elledge S.J. (2017). Genetic interrogation of replicative senescence uncovers a dual role for USP28 in coordinating the p53 and GATA4 branches of the senescence program. Genes Dev..

[65] Wang X., Liu Z., Zhang L., Yang Z., Chen X., Luo J., Zhou Z., Mei X., Yu X., Shao Z. (2018). Targeting deubiquitinase USP28 for cancer therapy. Cell Death Dis..

[66] Bolstad B.M., Irizarry R.A., Astrand M., Speed T.P. (2003). A comparison of normalization methods for high density oligonucleotide array data based on variance and bias. Bioinformatics.

[67] Yi H., Thurberg B.L., Curtis S., Austin S., Fyfe J., Koeberl D.D., Kishnani P.S., Sun B. (2012). Characterization of a canine model of glycogen storage disease type IIIa. Dis Model Mech.

