# Supplementary material for: Transcriptomic and Proteomic Analysis of Clear Cell Foci (CCF) in the Human Non-Cirrhotic Liver Identifies Several Differentially Expressed Genes and Proteins with Functions in Cancer Cell Biology and Glycogen Metabolism
Source: Molecules. 2020 Sep 10;25(18):4141. doi: 10.3390/molecules25184141 (PMC7570661; doi:10.3390/molecules25184141)
Supplement: Supplementary file 1 [file molecules-25-04141-s001.pdf]

Supplementary data file: Supplementary Figures and Tables

TITLE: Transcriptomic and Proteomic analysis of clear cell foci (CCF) in the human non-cirrhotic liver identifies several differentially expressed genes and proteins with functions in cancer cell biology and glycogen metabolism.

AUTHORS

Christoph Metzendorf[1,2], Katharina Wineberger[1], Jenny Rausch[1], Antonio Cigliano[1,3], Diego F. Calvisi[1,3], Kristin Peters[1], Baodong Sun[4], Daniela Mennerich[5,6], Thomas Kietzmann[5,6], Frank Dombrowski[1] and Silvia Ribback[1]

email-Addresses

christoph.metzendorf@igp.uu.se
katharina.wineberger@hotmail.de
antonio.cigliano@klinik.uni-regensburg.de
diego.calvisi@klinik.uni-regensburg.de
kristin.peters@med.uni-greifswald.de
baodong.sun@duke.edu
daniela.mennerich@oulu.fi
thomas.kietzmann@oulu.fi
frank.dombrowski@med.uni-greifswald.de
silvia.ribback@med.uni-greifswald.de

Affiliations

1 - Universitaetsmedizin Greifswald, Institut fuer Pathologie, Friedrich-Loeffler-Str. 23e, 17475 Greifswald, Germany

2 - current affilition
Uppsala University, Department of Immunology, Genetics and Pathology, BMC, Husargatan 3 751 24 Uppsala, Sweden

3 - current affiliation
Regensburg Universitaetsklinikum, Institut fuer Pathologie, Franz-Josef-Strauß-Allee 11, 93053 Regensburg, Germany

4 - Division of Medical Genetics, Department of Pediatrics, Duke University Medical Center, Durham, North Carolina 27710, USA

5 - Faculty of Biochemistry and Molecular Medicine, University of Oulu, Oulu, Finland.

6 - Biocenter Oulu, University of Oulu, Oulu, Finland.

Corresponding author:
Silvia Ribback

Universitaetsmedizin Greifswald
Institut fuer Pathologie
Friedrich-Loeffler-Str. 23e
17475 Greifswald, Germany

Phone: +493834865732
Fax: +493834865778

SUPPLEMENTARY FIGURES

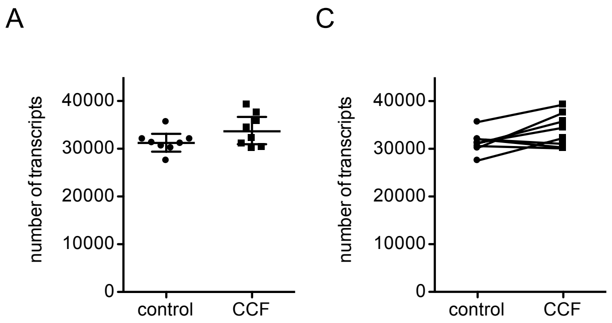

Supplementary Figure S1: (**A**) Number of transcripts above threshold per sample and sample-group (control and clear cell foci (CCF)); data of individual samples are shown as dots (controls) and squares (CCF) and geometric means and 95% confidence interval are indicated by lines. (**B**) Number of transcripts expressed above no-expression threshold per sample and sample-group (control and clear cell foci (CCF)); data of individual samples are shown as dots (controls) and squares (CCF) and data of each patient is connected by a line.

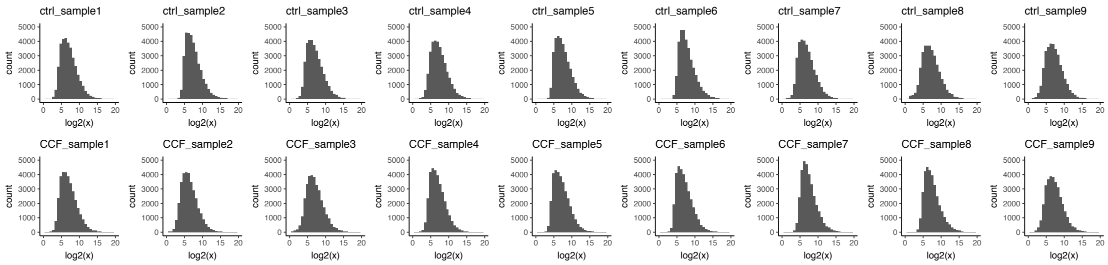

Supplementary Figure S2: The distribution of log2 transformed gene expression data approximates normal distribution in all samples and is similar between samples from the same patient. ctrl = control samples, CCF = clear cell foci samples, Sample1-Sample9 denote different patients.

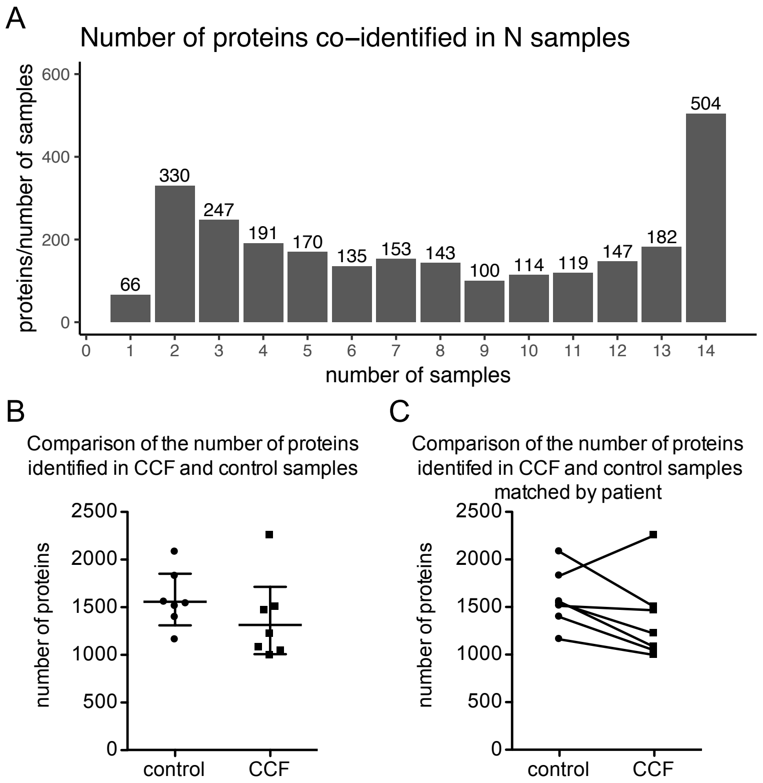

Supplementary Figure S1: (**A**) Number of proteins co-identified in the indicated number of samples. (**B**) Number of proteins identified per sample and sample-group (control and clear cell foci (CCF)); data of individual samples are shown as dots (controls) and squares (CCF) and geometric means and 95% confidence interval are indicated by lines. (**C**) Number of proteins identified per sample and sample-group (control and clear cell foci (CCF)); data of individual samples are shown as dots (controls) and squares (CCF) and data of each patient is connected by a line.

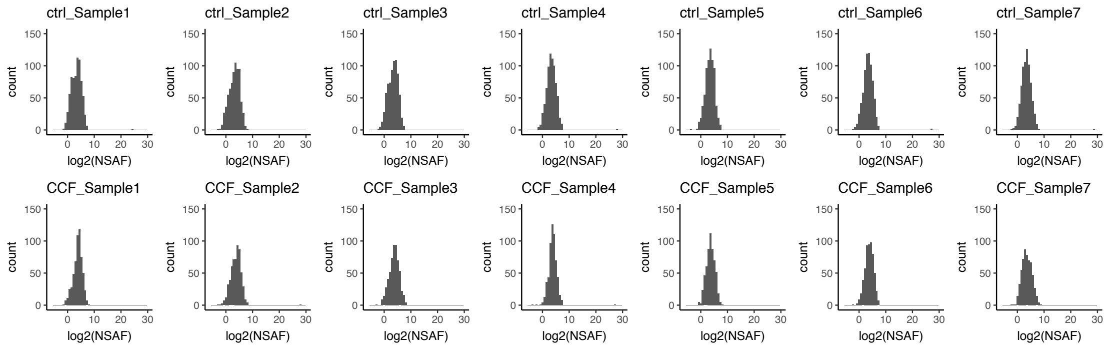

Supplementary Figure S2: The distribution of log2 transformed relative protein abundances (normalized spectral abundance factor (NSAF, materials and methods in main text)) approximates normal distribution in all samples and is similar between samples from the same patient. ctrl = control samples, CCF = clear cell foci samples, Sample1-Sample7 denote different patients.

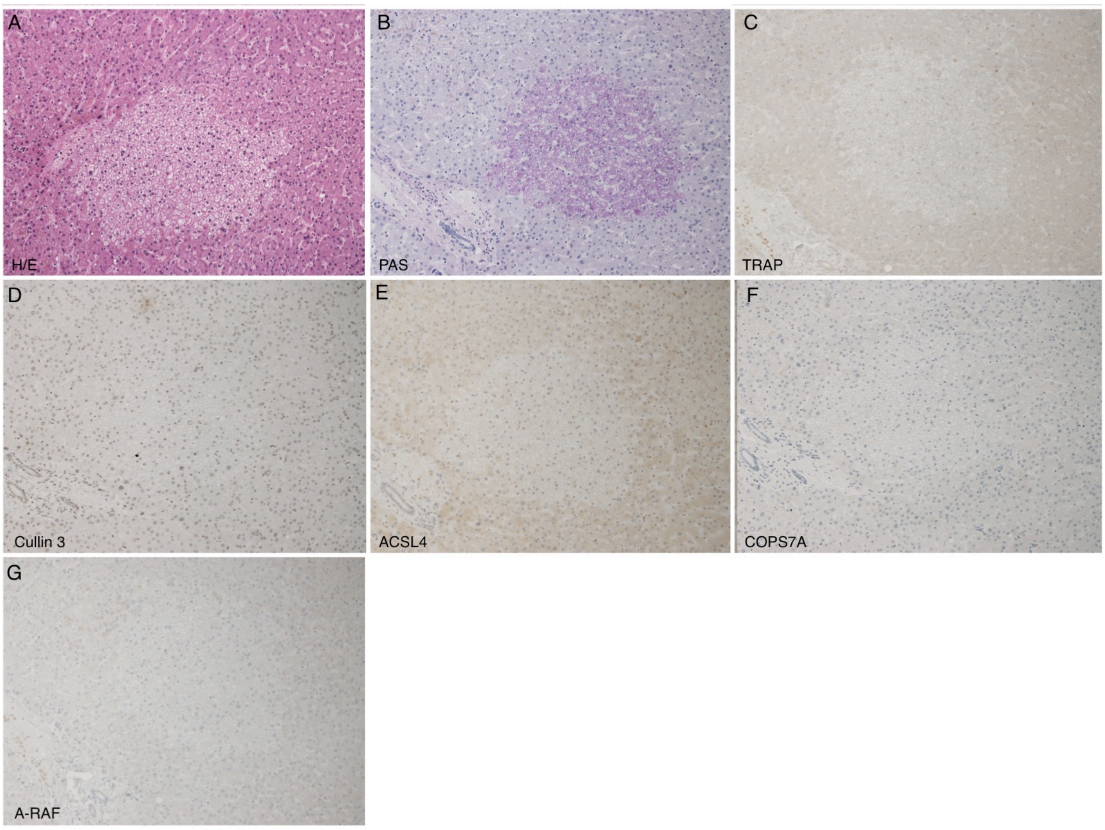

Supplementary Figure S3: Histochemistry and immunohistochemistry of representative human liver specimens with CCF and neighboring tissue. Hematoxilin/Eosin (H/E) staining (A), PAS reaction (B), and immunohistochemical detection of TRAP (C), Cullin 3 (D), ACSL4 (E), COPS7A (F), and A-RAF (G).

SUPPLEMENTARY TABLES

Table S1: RNA concentration and quality as determined by Nanodrop and Bioanalyzer respectively

Sample	RNA concentration (ng/µl)	RNA yield (ng)	RIN
sample 1 - H	23	688	7,1
sample 1 - N	24	717	7,9
sample 2 - H	25	744	7,2
sample 2 - N	29	862	7,1
sample 3 - H	24	725	7,3
sample 3 - N	20	615	7,2
sample 4 - H	50	1514	6,9
sample 4 - N	36	1081	6,3
sample 5 - H	24	717	7,1
sample 5 - N	28	1120	7,0
sample 6 - H	17	520	6,9
sample 6 - N	23	705	6,9
sample 7 - H	26	765	7,2
sample 7 - N	26	793	7,2
sample 8 - H	20	609	6,4
sample 8 - N	22	645	6,4
sample 9 - H	16	470	6,8
sample 9 - N	25	738	6,3
sample 10 - H	44	1760	8,5
sample 10 - N	66	2640	6,2

Table S2: Antibodies used for immunohistochemistry

Protein	product # / [reference]	company / source	antigen unmasking	antibody dilution
ACSL4	HPA005552	SigmaAldrich / Atlas Antibodies	boiling, pH 6	1:100
Cullin 3	342200	Life technologies	boiling, pH 6	1:100
COPS7A	HPA026915	SigmaAldrich / Atlas Antibodies	boiling, pH 6	1:100
CYP5B	15469-1-AP	ProteinTech	boiling, pH 6	1:100
HSP10	HPA038755	SigmaAldrich /Atlas Antibodies	boiling, pH 6	1:1000
LGAL7	sc-271473	Santa Cruz Biotechnology	boiling, pH 6	1:50
monad/WDR92	[1]	Makio Saeki<br>Division of Dental Pharmacology<br>Niigata University Graduate School of Medical and Dental Sciences<br>2-5274 Gakkocho-dori, Chuo-ku,<br>Niigata, 951-8514, Japan	boiling, pH 6	1:100
RAB12	HPA040727	SigmaAldrich / Atlas Antibodies	boiling, pH 6	1:100
RAB35	HPA054146	SigmaAldrich / Atlas Antibodies	boiling, pH 6	1:100
A-RAF	HPA066326	SigmaAldrich /Atlas Antibodies	boiling, pH 6	1:100
STBD1	HPA011952	SigmaAldrich / Atlas Antibodies	boiling, pH 6	1:100
TRAP 1	HPA041082	SigmaAldrich / Atlas Antibodies	boiling, pH 6	1:100
USP28	HPA006778	SigmaAldrich / Atlas Antibodies	boiling, pH 6	1: 50

Literature references

1. Saeki M, Irie Y, Ni L, Yoshida M, Itsuki Y, Kamisaki Y. Monad, a WD40 repeat protein, promotes apoptosis induced by TNF-alpha. Biochem Biophys Res Commun. 2006; 342: 568–72. doi: 10.1016/j.bbrc.2006.02.009.
